# Effect of pore diameter and length on electrochemical CO_2_ reduction reaction at nanoporous gold catalysts†

**DOI:** 10.1039/d1sc05743j

**Published:** 2022-02-22

**Authors:** Akansha Goyal, Christoph J. Bondue, Matthias Graf, Marc T. M. Koper

**Affiliations:** Leiden Institute of Chemistry, Leiden University PO Box 9502 2300 RA Leiden The Netherlands m.koper@chem.leidenuniv.nl; Faculty of Chemistry and Biochemistry, Ruhr University Bochum Bochum D-44780 Germany

## Abstract

In this work, we employ differential electrochemical mass spectrometry (DEMS) to track the real-time evolution of CO at nanoporous gold (NpAu) catalysts with varying pore parameters (diameter and length) during the electrochemical CO_2_ reduction reaction (CO_2_RR). We show that due to the increase in the local pH with increasing catalyst roughness, NpAu catalysts suppress the bicarbonate-mediated hydrogen evolution reaction (HER) compared to a flat Au electrode. Additionally, the geometric current density for CO_2_RR increases with the roughness of NpAu catalysts, which we attribute to the increased availability of active sites at NpAu catalysts. Together, the enhancement of CO_2_RR and the suppression of competing HER results in a drastic increase in the faradaic selectivity for CO_2_RR with increasing pore length and decreasing pore diameter, reaching near 100% faradaic efficiency for CO in the most extreme case. Interestingly, unlike the geometric current density, the specific current density for CO_2_RR has a more complicated relation with the roughness of the NpAu catalysts. We show that this is due to the presence of ohmic drop effects along the length of the porous channels. These ohmic drop effects render the pores partially electrocatalytically inactive and hence, they play an important role in tuning the CO_2_RR activity on nanoporous catalysts.

## Abstract 2

In this work, we employ differential electrochemical mass spectrometry (DEMS) to track the real-time evolution of CO at nanoporous gold (NpAu) catalysts with varying pore parameters (diameter and length) during the electrochemical CO_2_ reduction reaction (CO_2_RR).

## Graphical abstract

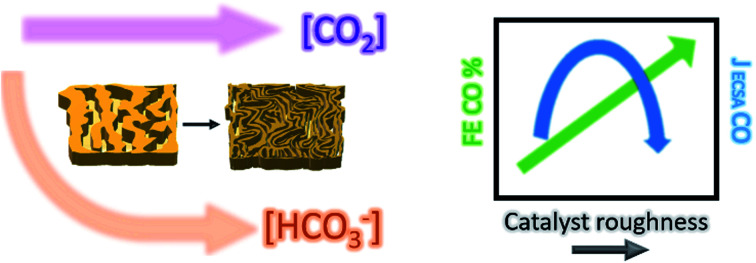

## 1. Introduction

Electrochemical reduction of CO_2_ (CO_2_RR) can be used to achieve a carbon-neutral energy cycle wherein carbon-based fuels can be produced with net zero emissions by using renewable electricity.^1–3^ However, at present, the economic feasibility of this reaction remains an issue, primarily due to its low energy efficiency in commonly employed bicarbonate electrolytes, especially at high current densities.^4,5^ Consequently, significant research efforts have been made towards optimizing the catalyst design as well as the reaction process conditions for achieving better CO_2_RR selectivities.^6–9^

Recently, meso- or nanoporous electrocatalyst materials have emerged as an interesting strategy to tune the selectivity of CO_2_RR. To this end, most of the research has focused on the effect intrinsic properties of nanoporous structures (such as high, potentially active surface area, high relative density of stepped sites and grain boundaries) have on CO_2_RR activity.^10–15^ However, the effect of porosity towards tuning the local reaction environment by regulating the near-surface concentration of different reactive species (such as CO_2_, HCO_3_^−^ and OH^−^) has received less scrutiny. Given that local concentration gradients play a significant role in tuning the competition between CO_2_RR and the parasitic hydrogen evolution reaction (HER) on flat polycrystalline electrodes,^8,16,17^ it can be expected that the mass transport limitations introduced by confinement effects in and around the nanoporous channels will also affect the competition between these two reactions.^18–20^

In this respect, some recent studies have indeed emphasized the importance of local diffusional gradients in tuning the CO_2_RR activity on nanoporous electrodes.^21–25^ In general, it has been shown that with the increasing roughness/thickness of the nanoporous catalysts, the local pH at the surface also increases, resulting in the suppression of bicarbonate-mediated HER reaction (HCO_3_^−^ + 2e^−^ → H_2_ + 2CO_3_^2−^). However, conflicting results have been obtained on the effect of local concentration gradients in tuning the rate of CO_2_RR reaction on nanoporous catalysts. Surendranath and co-workers have shown the formation rate of CO on nanoporous gold (NpAu) is largely independent of the catalyst's pore dimensions, which they attributed to negligible concentration gradient for CO_2_ between the bulk environment to the surface due to its slow acid–base equilibria in the electrolyte.^22,26^ However, in the case of nanoporous silver catalysts, the same authors observe that increasing catalyst layer thickness results in higher partial current densities for CO_2_RR.^21^ Meanwhile, Atwater and co-workers have observed a similar enhancement in the rate of CO_2_RR in the case of NpAu catalysts, which they attributed to the presence of grain boundaries on nanoporous electrodes.^24^ On the other hand, in a similar study, Cheng and co-workers have found the opposite effect of catalyst size on CO_2_RR and attributed this to the decreasing solubility of CO_2_ with increasing local pH at the surface.^25^

One of the complications in unambiguously elucidating the role of nanoporous catalysts in tuning the activity of the electrocatalytic reactions (CO_2_RR and HER) arises from the ancillary participation of homogenous acid–base equilibria (CO_2_(aq.) + OH^−^ ↔ HCO_3_^−^; HCO_3_^−^ + OH^−^ ↔ CO_3_^2−^ + H_2_O) in regulating the local concentration gradients at the catalyst surface.^27–29^ Consequently, different concentration gradients can be generated near similar catalysts due to the convoluted interplay between the homogenous reactions, mass transport, and the heterogeneous reactions, especially in the case of long-term bulk electrolysis measurements.^30^ Hence, in order to more reliably disentangle these effects, it is imperative to track the real-time evolution of the reaction products as well as the local concentration gradients by using an online detection technique. Moreover, cell geometries with well-defined mass transport conditions are also desirable in order to avoid the time-dependent concentration polarization effects.^31^ To circumvent these issues, we have recently developed two online methods, namely, a gold ring based rotating ring disk electrode (RRDE) technique and an online differential electrochemical mass spectrometry (DEMS) technique based on a dual thin-layer flow cell, for the quantitative detection of CO during CO_2_RR under well-defined mass transport conditions.^8,17,32,33^

In this paper, we present a study on the role of pore parameters (diameter and length) in tuning the competition between CO_2_RR and HER on nanoporous Au (NpAu) catalysts by using our DEMS technique. We find that with decreasing pore diameter and increasing pore length the faradaic selectivity for CO_2_RR increases dramatically (up to ∼100%), even under sub-optimal reaction conditions (0.5 atm. CO_2_ in 0.1 M NaHCO_3_ plus 0.4 M NaClO_4_). We show that this enhancement arises due to two factors: (i) the suppression of bicarbonate mediated HER reaction with increasing local pH at the catalyst surface and (ii) the enhancement of CO_2_RR at NpAu catalysts due to the increased availability of active sites with increasing catalyst roughness. Moreover, we show that the ohmic drop effects across the porous channels play an important role in tuning the obtained specific current densities for CO_2_RR as they render the pores partially inactive towards electrocatalysis. Hence, by using the online DEMS technique, we elucidate the convoluted interplay between catalyst geometry effects and local concentration gradient effects in tuning the activity/selectivity of CO_2_RR at nanoporous catalysts.

## 2. Experimental section

### 2.1 Chemicals

The electrolytes were prepared from H_2_SO_4_ (98% by wt. solution, EMSURE, Merck), NaHCO_3_ (≥99.7%, Honeywell Fluka), NaClO_4_ (99.99%, trace metals basis, Sigma-Aldrich), NaH_2_PO_4_ (≥99.0%, EMSURE, Merck), Na_2_HPO_4_ (≥99.0%, EMSURE, Merck) and Ultrapure water (MilliQ gradient, ≥18.2 MΩcm, TOC <5 ppb). CO (4.7 purity, Linde), Ar (6.0 purity, Linde) and CO_2_ (4.5 purity, Linde) were used for purging the electrolytes. To prepare the nanoporous Au (NpAu) catalysts, KAu(CN)_2_ (99.95%, trace metals basis, Sigma-Aldrich), KAg(CN)_2_ (Sigma-Aldrich), Na_2_CO_3_ (≥99.5%, Sigma-Aldrich) and HClO_4_ (60% by wt. solution, EMSURE, Merck) were used.

### 2.2 NpAu catalyst synthesis and characterization

Before each experiment, the substrate electrode (polycrystalline Au; geometric area = 0.78 cm^2^) was mechanically polished on Buehler micro-polishing cloth (8 inches) with decreasing sizes of diamond polishing suspension, namely, 3 μm, 1 μm and 0.25 μm. Next, it was sonicated in ultrapure water and acetone for 10 minutes to remove any organic/inorganic impurities. Thereafter, the NpAu catalyst layer was formed on the substrate electrode by following the electrochemical alloying-dealloying procedure outlined previously.^34–36^ Briefly, a binary alloy solution of 5 : 1 ratio of Ag/Au was prepared with KAg(CN)_2_ and KAu(CN)_2_ in 0.25 M Na_2_CO_3_ buffer solution and it was potentiostatically electrodeposited onto the flat Au substrate electrode at −1.2 V (*vs.* Ag/AgCl) for 9 min. Thereafter, Ag was de-alloyed from the film by electrochemically cycling (20 cycles) the electrode in 1 M HClO_4_ solution between 1.05 V to 1.2 V (*vs.* Ag/AgCl) at 5 mV s^−1^. The as-prepared NpAu catalyst (referred to as “NpAu4”) was thermally coarsened to obtain varying pore diameter and height, at 523.15 K, for 120 s, 60 s and 30 s to form NpAu1, NpAu2 and NpAu3, respectively. To avoid faulty activity measurements due to any residual CO_2_ in the pores, a new catalyst layer was prepared for each measurement. To determine the roughness factor of the catalysts, electrochemical characterization was performed in 0.1 M H_2_SO_4_ (0.05 V to 1.75 V *vs.* RHE at a scan rate of 50 mV s^−1^) to obtain the electrochemically active surface area (ECSA) of the catalyst by calculating the charge from the reduction peak for the Au oxide in the characterization CV and dividing it by the specific charge of one monolayer of Au (390 μC cm^−2^).^37^

The morphology and the composition of the different NpAu catalysts was checked by using the ETD detector (for SEM imaging) and the EDS detector (for Energy Dispersive X-ray Spectroscopy) of Apreo SEM (Thermo Scientific), respectively. After the electrochemical measurements, the NpAu samples were rinsed with MilliQ water and upon drying placed on a custom-made SEM holder for analysis.

### 2.3 Differential electrochemical mass spectrometry (DEMS) measurements

All the electrochemical measurements were performed with a home-built DEMS set-up (described in detail elsewhere).^32^ The ionization potential of the ion source of the mass spectrometer was set to −27.5 V (*vs.* the potential of the formation room and +72.5 V *vs.* ground) to circumvent the fragmentation of CO_2_ at mass 28 (CO^+^) which allowed for the ionic signal at mass 28 to be used for the quantitative detection of CO formed during CO_2_RR.^32^ To achieve well-defined mass transport conditions during the CO_2_RR studies, a dual thin layer cell with a flow cell geometry was used (described in detail elsewhere).^38^ We note here that for all the CO_2_RR measurements, instead of using CO_2_ saturated conditions, we employed a 1 : 1 (vol.) ratio of Ar/CO_2_. This was done in order to prevent bubble attachment in the microfluidic channels of the dual thin layer cell, which becomes unavoidable when high concentration of hydrophobic CO_2_ is dissolved in the electrolyte, as it tends to salt out in the thin channels of the cell body (made of Kel-F) over the course of the measurements.^17^ Hence for all the CO_2_RR measurements, an Ar/CO_2_ saturated electrolyte (0.1 M NaHCO_3_ plus 0.4 M NaClO_4_ or 0.1 M NaHCO_3_ plus 2 M NaClO_4_) was introduced into the top (electrochemical) compartment of the cell where the working electrode (NpAu or Flat Au) was placed. For all the CO_2_RR studies, the cyclic voltammograms were recorded between −0.75 V and −1.2 V *vs.* normal hydrogen electrode (NHE; where a Ag/AgCl reference was used in all the measurements and the potentials were converted to the NHE scale afterwards) at a scan rate of 5 mV s^−1^. The flow of the electrolyte (5 μL s^−1^) through the cell was achieved with a syringe pump which swept the electrolyte (along with the electrochemical products and the unreacted analyte) from the top compartment to the bottom compartment of the cell which was interfaced with the vacuum of the mass spectrometer with the help of a porous Teflon membrane and a porous steel frit. The products of HER and CO_2_RR were detected at the ionic signal at *m*/*z* 2 (H_2_) and *m*/*z* 28 (CO), respectively. The ionic current obtained with the DEMS is related to the molar flux of the species by eqn 1:1
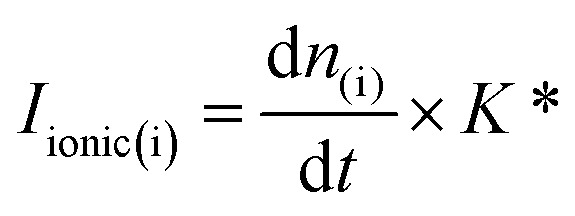
where, *I*_ionic(i)_ is the ionic current due to a species i, d*n*_(i)_/d*t* is the molar flux of i into the mass spectrometer and K* is a proportionality constant that reflects the sensitivity of the DEMS setup for i, which among other things is dependent on the ionization probability of i.^39^ Here, the molar flux 
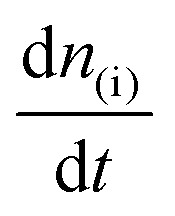
 is associated to the faradaic current *I*_Faradaic(i)_ by Faraday's law:2
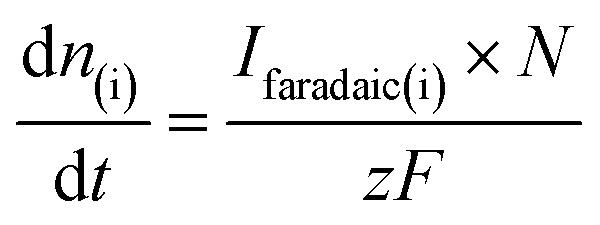
where *N* is the transfer efficiency of species i in a given cell geometry/assembly, *z* is the number of electrons involved in the electrochemical reaction and *F* is Faraday's constant. By substituting eqn (2) into eqn (1), we obtain:3
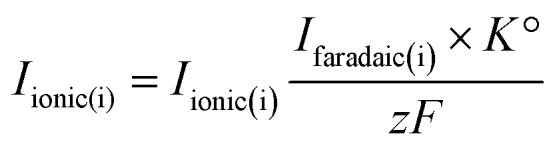
where *K*° = K* × *N*° is the cell calibration constant. In order to quantify the ionic signals, in every new cell assembly, calibration measurements need to be performed to determine the value of the calibration constant (*K*°) for H_2_ and CO, respectively. For the calibration of H_2_, measurements were performed under Ar saturated conditions with a blank electrolyte (0.1 M phosphate buffer and 0.4 M NaClO_4_), as shown in Fig. S1a of the ESI,† where H_2_ was evolved with 100% faradaic efficiency (scan window of −0.6 V to −1.8 V *vs.* Ag/AgCl at a scan rate of 5 mV s^−1^). Using eqn (3), *K*^°^_H2_ was determined 
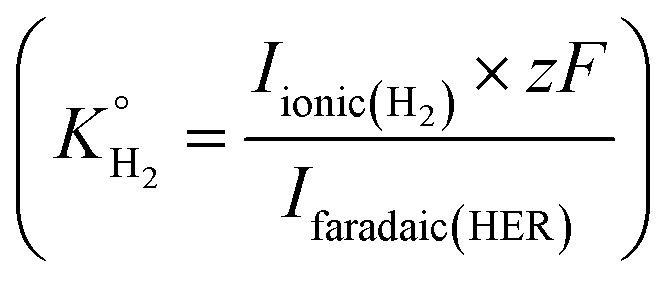
. For the calibration of CO, bulk CO oxidation was performed under CO saturated conditions with a blank electrolyte (0.1 M phosphate buffer and 0.4 M NaClO_4_), as shown in Fig. S1b† of the ESI,† where CO_2_ was evolved with 100% faradaic efficiency (scan window of −0.6 V to 0.5 V *vs.* Ag/AgCl at a scan rate of 5 mV s^−1^). Using eqn (3), *K*^°^_CO_ was determined 
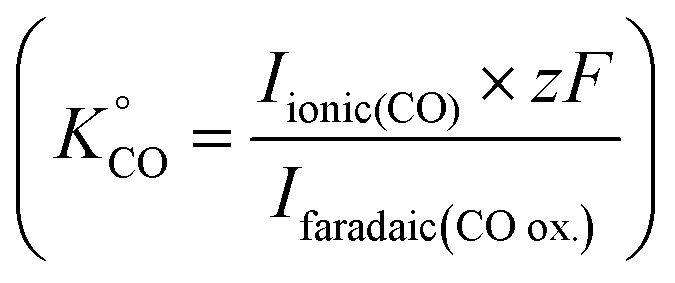
. Next, the ionic signals obtained at mass 2 and mass 28 during CO_2_RR measurements could be used to calculate the partial current densities for H_2_ and CO formation from eqn (3).

The faradaic selectivity for CO_2_RR and HER could then be calculated as follows:4
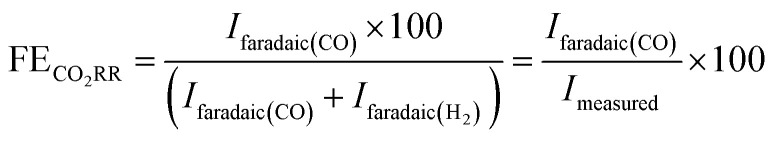
and,5
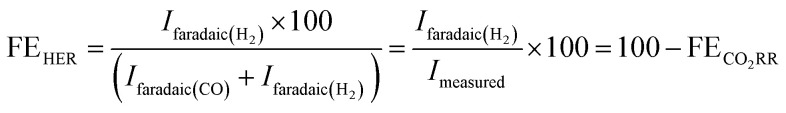


In summary, by determining the calibration constants (*K*^°^_H2_ and *K*^°^_CO_), the online DEMS technique can be used for quantitatively studying CO_2_RR on NpAu catalysts.

## 3. Results and discussion

In this work we studied the role of pore parameters (diameter and length) in tuning the activity/selectivity of electrochemical CO_2_ reduction reaction (CO_2_RR). For this we prepared four different nanoporous Au (NpAu) samples *via* an electrochemical alloying-dealloying and subsequent thermal coarsening procedure, as outlined in Section 2.2. This resulted in the formation of typical bi-continuous porous structure of ligaments and pores in all the NpAu samples, as confirmed by the scanning electron microscopy (SEM) images (see Fig. S3 in the ESI†). Moreover, the SEM images also confirm that the thermal coarsening of NpAu samples leads to the step wise decrease of the ligament diameter of the porous network. Similar to our previous study,^34^ the pore diameters change roughly from 40 nm to 10 nm in going from the least coarsened sample to most coarsened sample *i.e.* NpAu1 to NpAu_4_. Moreover, electrochemical characterization (see Fig. S2 in the ESI†) of the samples also shows a systematic change, such that the electrochemically active surface area (ECSA) of the catalysts decreases with increasing coarsening.

Thus, in going from NpAu1 to NpAu_4_ the pore diameter decreases and the pore length increases. This in turn leads to decreasing “effective diffusion” through the porous channels due to the generation of additional diffusional gradients along the length of the pores. This has already been studied in detail by the means of scanning electrochemical microscopy (SECM) where it was shown that the effective diffusion coefficient of any species (*D*_eff_ = *P*′*D*; where *P*′ is porosity of the catalyst corrected by its tortuosity *τ* and *D* is the diffusion coefficient of the species in the solution phase) increases with increasing coarsening of the NpAu catalysts.^40^

**Fig. 1:**
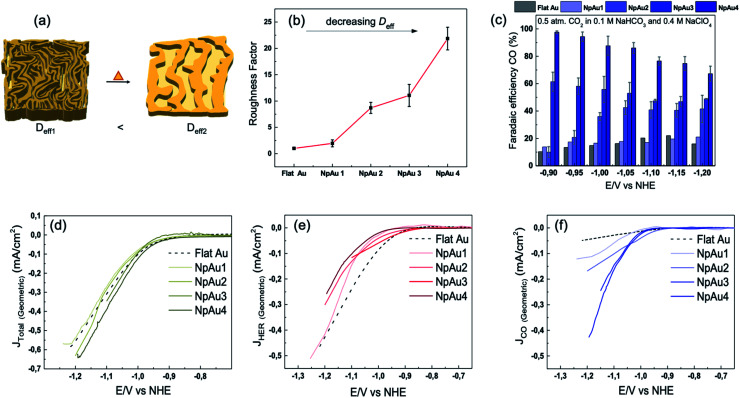
(a) Schematic representation of NpAu catalysts illustrating how thermal coarsening results in the self-similar growth of the NpAu ligaments such that the pore diameter increases and the effective pore length decreases with increasing coarsening. This also results in a decreasing effective diffusive flow through the porous channels. (b) Roughness factors of different Au catalysts as determined by the ECSA values obtained from Fig. S2 (ESI†), where *D*_eff_ decreases with increasing roughness. (c) faradaic efficiency for CO formation on the different Au catalysts at different potentials (*vs.* NHE), as obtained from the DEMS measurements by using eqn (4). (d) Total geometric current density on different Au catalysts during CO_2_RR, (e) Partial geometric current density for HER on different Au catalysts obtained from the ionic current at *m*/*z* 2 by using eqn (3) and (f) partial geometric current density for CO formation on different Au catalysts obtained from the ionic current at *m*/*z* 28 by using eqn (3). All the measurements were done in 0.5 atm. CO_2_, in 0.1 M NaHCO_3_ plus 0.4 M NaClO_4_ containing electrolyte with a scan rate of 5 mV s^−1^ and a flow rate of 300 μL min^−1^.

In Fig. 1b, we show the roughness factor of the different NpAu catalysts with respect to the polycrystalline Au electrode (Flat Au), calculated by using the as-determined ECSA (shown in Fig. S2 in the ESI†) of the different surfaces. As expected, the roughness factor of the catalyst surface increases with decreasing thermal coarsening. We note here that in all the NpAu samples, we detect around 7 atomic-% of residual Ag with EDX spectroscopy (shown in Fig. S3 in the ESI†).

Thereafter, we studied CO_2_RR at these catalysts by using the online DEMS, where the ionic signals at *m*/*z* 2 and 28 were used to quantify the amount of H_2_ and CO, respectively, evolved during CO_2_RR on the different catalyst surfaces (for further details see Section 2.3). In Fig. 1c, we show the faradaic efficiency for CO_2_RR on the different Au catalysts as derived from the DEMS measurements by using eqn (4). In agreement with the previous studies on nanoporous Au catalysts,^17,22,24^ we see that the faradaic efficiency for CO_2_RR increases with increasing roughness factor of the catalysts, *i.e.* with decreasing pore diameter and increasing pore length (Flat Au < NpAu_1_ < NpAu_2_ < NpAu_3_ < NpAu_4_). Remarkably, in the case of NpAu_4_, we achieve ∼100% faradaic efficiency for CO_2_RR even with 0.5 atm. of CO_2_. Here, the sub-optimal reaction conditions for CO_2_RR (0.5 atm. of CO_2_ instead of 1 atm. CO_2_) also explain the uncharacteristically low faradaic efficiency for CO in the case of flat polycrystalline Au (Flat Au) catalyst. We note that in addition to the low partial pressure of CO_2_, the high Na^+^ ion concentration in the electrolyte also contributes to a lower faradaic selectivity towards CO_2_RR. We have shown previously with our RRDE technique that with increasing cation concentration in the electrolyte (at a fixed bicarbonate concentration) the faradaic efficiency towards CO_2_RR decreases on flat Au polycrystalline electrodes.^8,33^ In that work we achieved faradaic selectivities close to 60% in 1 atm. CO_2_ with 0.5 M Na^+^ ion containing electrolytes.^33^ Hence, the faradaic efficiency of around 20% in the case of flat Au catalysts (as shown in Fig. 1c) can be attributed to the combination of low CO_2_ partial pressure (0.5 atm.) and a high cation concentration in the electrolyte (0.5 M) in our measurements.

In Fig. 1d, we plot the experimentally measured total geometric current density, and in Fig. 1e and f we plot the corresponding partial geometric current densities for HER and CO_2_RR calculated from the ionic currents measured at mass 2 and 28, respectively. We see that the geometric current density for HER decreases in going from Flat Au to NpAu_4_ (as shown in Fig. 1e) and hence, the enhancement in the CO_2_RR faradaic efficiency with the increasing roughness of the NpAu catalysts can, at least in part, be attributed to the suppression of competing HER reaction.^21,22,25^ We have shown previously with the RRDE technique^8,33^ that in bicarbonate containing electrolytes, HER can either be mediated by HCO_3_^−^ ions (eqn (6)) or by H_2_O molecules (eqn (7)):

6 HCO_3_^−^ + 2e^−^ → H_2_ + 2CO_3_^2−^

7 H_2_O + 2e^−^ → H_2_ + OH^−^

and that these two branches show a divergent dependence on electrolyte pH. It was observed that while HER due to the discharge of HCO_3_^−^ ions decreased with increasing pH, for water reduction reaction the opposite takes place.^8,41^ Moreover, the branch of HER that competes with CO_2_RR is largely dependent on the identity of the electrolyte that is employed in the measurements. In brief, the dominating branch of HER switches from water-mediated to HCO_3_^−^-mediated with increasing bicarbonate/cation concentration in the electrolyte.^8,33^ In accordance with these results, under the experimental conditions of our measurements (0.1 M NaHCO_3_ plus 0.4 M NaClO_4_) we expect bicarbonate mediated HER to be the main branch of hydrogen evolution that competes with CO_2_RR.

Moreover, we expect the partial current due to HER to be sensitive to the local pH which, among other things, is determined by the morphology of the electrode surface. In the case of nanoporous catalysts, with increasing catalyst roughness, the effective diffusion through the porous channels will decrease. As a result, the local pH will increase at the catalyst surface due to the hindered mass transport of locally generated OH^−^ ions away from the electrode and at the same time, due to the hindered mass transport of HCO_3_^−^ ions and CO_2_ (aq.) to the surface. Hence, we attribute the decreasing HER activity with increasing catalyst roughness, as observed in Fig. 1e, to the corresponding increase in local pH in going from Flat Au to NpAu_4_. This is because the increasing local alkalinity accelerates the homogeneous consumption of bicarbonate near the electrode surface:

8 HCO_3_^−^ + OH^−^ ↔ CO_3_^2−^ + H_2_O

which in turn leads to a paucity of HCO_3_^−^ ions for participating in the HER reaction.^8^ This is also in agreement with our previous study where we have showed that HER has a positive reaction order in bicarbonate concentration.^33^ As a result, a decreasing concentration of bicarbonate ions at the catalyst surface, due to their reaction with OH^−^, has detrimental effect on HER activity under these conditions. To further corroborate this, we made a crude estimation of surface concentration of hydroxyl ions as well as bicarbonate ions as a function of catalyst porosity/roughness in Section S2 of the ESI.† In agreement with our hypothesis, we see that the surface pH becomes more alkaline with increasing catalyst roughness (see Fig. S5a in the ESI†). This results in a corresponding decrease in the surface concentration of bicarbonate ions (see Fig. S5b in the ESI†). Moreover, the calculations show that even though the surface concentration of bicarbonate ions is decreasing with increasing catalyst roughness, they are not entirely depleted at the surface under the experimental conditions of our measurements. Nevertheless, this decrease in the surface concentration of bicarbonate ions leads to the suppression of theoretically calculated HER current density (Fig. S6 in the ESI†), thus showing a qualitative agreement with our experimental results.

**Fig. 2:**
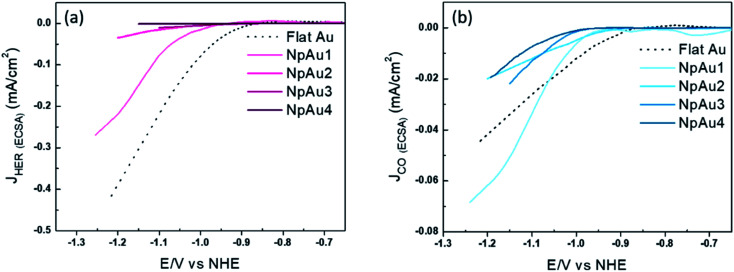
(a) Partial specific current density for HER on different Au catalysts (obtained from the data used in Fig. 1e and b). (b) Partial specific current density for CO formation on different Au catalysts (obtained from the data used in Fig. 1f). All the measurements were done in 0.5 atm. CO_2_, in 0.1 M NaHCO_3_ plus 0.4 M NaClO_4_ containing electrolyte with a scan rate of 5 mV s^−1^ and a flow rate of 300 μL min^−1^.

We also note here that the partial current density for HER decreases both with respect to the geometric surface area (Fig. 1e) and with respect to the ECSA of the catalysts (Fig. 2a).

On the other hand, for CO_2_RR, we see that the partial geometric current density increases with increasing pore length and decreasing pore diameter *i.e.* with increasing catalyst roughness, as shown in Fig. 1f. This is partially counter-intuitive since increasing local pH at the catalyst surface should also lead to a corresponding increase in the homogeneous consumption of CO_2_:

9 CO_2_(aq.) + OH^−^ ↔ HCO_3_^−^

Hence, similar to HER, we would expect that increasing local pH due to increasing catalyst roughness will result in the suppression of CO_2_RR. This would also be expected since CO_2_ (aq.) (*D*_CO_2__ = 1.6 × 10^−5^ cm^2^ s^−1^) and HCO_3_^−^ ions (*D*_HCO_3_^−^_ = 1.2 × 10^−5^ cm^2^ s^−1^) have similar diffusion coefficients.^30^ However, we see the opposite trend in Fig. 1f. This can be rationalized by the fact that even though CO_2_ and HCO_3_^−^ have similar diffusion coefficients, the rate of their homogenous reaction with the hydroxyl ions is drastically different. In fact, the rate of homogeneous CO_2_ consumption (*k*_9_^+^ = 2.23 × 10^3^ kg mol^−1^ s^−1^) is six orders of magnitude slower than the rate of homogeneous HCO_3_^−^ consumption (*k*_8_^+^ = 6 × 10^9^ kg mol^−1^ s^−1^).^27,42^ Consequently, the mass transport rate of CO_2_ (aq.) to the interface can outpace the rate of its homogeneous reaction, especially under the conditions of convection control (*e.g.* rotation control, flow control).

**Fig. 3:**
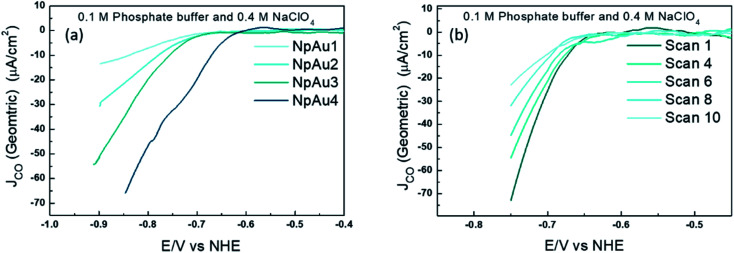
(a) Partial geometric current density for CO formation on different Au catalysts (Flat Au, NpAu_1_, NpAu_2_, NpAu_3_ and NpAu_4_) where the dual thin layer cell (along with the catalysts) was first flushed with a CO_2_ sat. 0.1 M Phosphate buffer plus 0.4 M NaClO_4_ containing electrolyte; the measurements were done in Ar sat. conditions (with the same electrolyte) after flushing the cell with Ar sat. electrolyte for 10 min. and in (b) the 10 subsequent scans under the same experimental conditions described above on NpAu4 catalyst. Scan rate: 5 mV s^−1^ and flow rate: 300 μL min^−1^.

To validate this further, we performed measurements where the different NpAu catalysts were first exposed to a CO_2_-saturated electrolyte and thereafter, currents due to CO formation were measured under Ar-saturated conditions (shown in Fig. 3). Interestingly, we see that all the NpAu catalysts show appreciable currents due to CO formation even in the absence of a continuous CO_2_ supply (Fig. 3a). Moreover, these currents persist after ten subsequent scans (Fig. 3b) even on the catalyst with the highest roughness factor (NpAu_4_). Together, these results show that despite the increasing mass transport limitations and with this an increasing local alkalinity in the porous channels, NpAu catalysts are able to furnish an appreciable reservoir of CO_2_. Moreover, the local CO_2_ reservoir is able to take part in CO_2_RR without completely getting homogeneously consumed. In fact, with increasing catalyst roughness we obtain higher currents for CO formation (Fig. 3a), thus indicating that with increasing pore length we generate a larger CO_2_ reservoir at the catalyst surface.

Hence, the slow homogeneous reaction of CO_2_ explains why the CO_2_RR current density does not decrease in going from Flat Au to NpAu_4_. On the other hand, the availability of electrochemically active sites at the catalyst surface increases with increasing catalyst roughness, as a higher surface roughness automatically comes with more surface defects which are commonly considered sites of increased reactivity. Together, the increase in the number of active sites and/or increased surface defects explains the observed enhancement in CO_2_RR current density with increasing catalyst roughness.

Interestingly, unlike HER, we see diverging trends when the partial geometric current density (current with respect to the geometric surface area) and partial specific current density (current with respect to the ECSA) for CO_2_RR are compared. In Fig. 2b we plot the partial specific current density for CO_2_RR and we see that while it increases in going from Flat Au to NpAu1, a further increase in the catalyst roughness results in a lower specific current density for CO_2_RR, with no clear trend between NpAu_2_ to NpAu_4_. Given that the CO_2_RR activity is tied to the availability of active sites at the catalyst surface, the fact that it scales with the geometric surface area (Fig. 1f) but does not scale with the ECSA (Fig. 2b) suggests that not the entire surface of the NpAu catalysts participates in electrocatalysis.

**Fig. 4:**
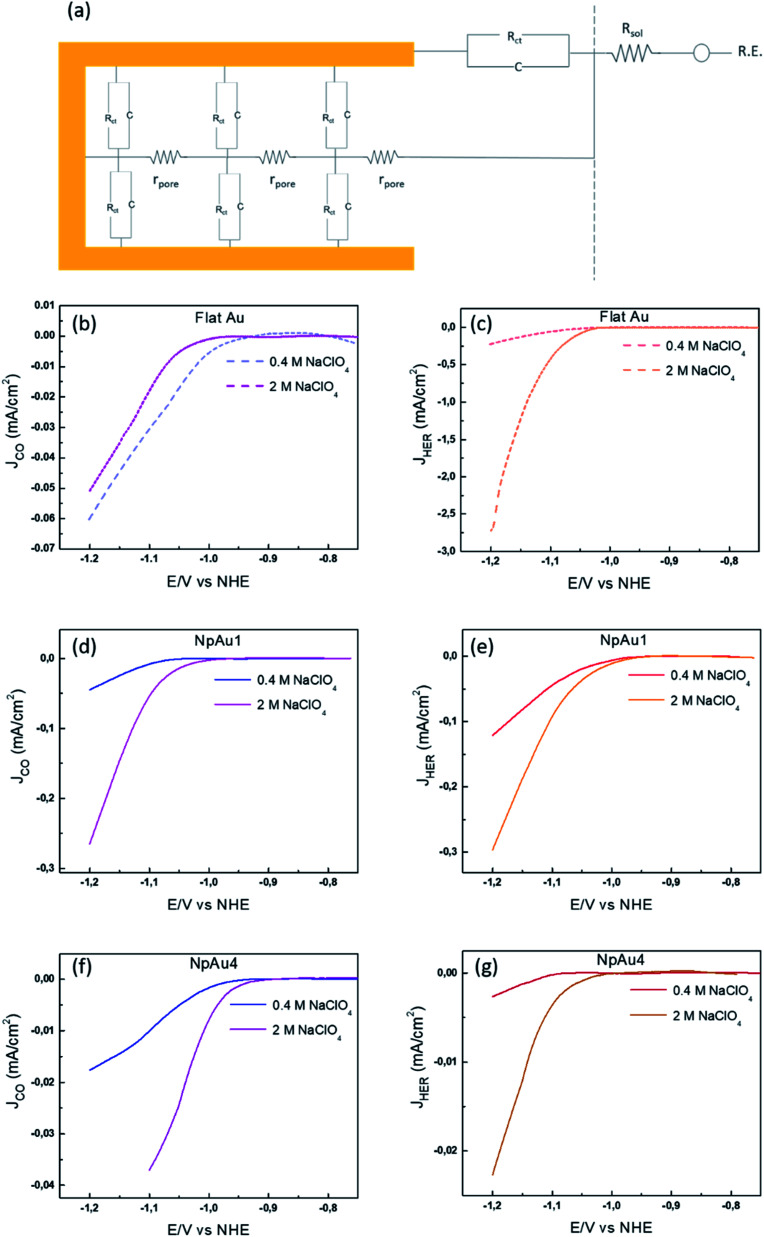
(a) Schematic representation of the circuit at NpAu catalysts where *R*_sol_ is the solution resistance between the reference electrode and the working electrode, *R*_ct_, *C* are the distributed charge transfer resistance and the distributed capacitance associated with the faradaic reaction at the catalyst surface and *r*_pore_ is the distributed resistance of the pore per unit length and per unit surface area of the porous film. Partial specific current density for CO formation as obtained from the ionic current at *m*/*z* 28 by using eqn (3) with 0.5 atm. CO_2_ in 0.1 M NaHCO_3_ plus 0.4 M NaClO_4_ containing electrolyte (blue) and in 0.1 M NaHCO_3_ plus 2 M NaClO_4_ containing electrolyte (pink) on (b) Flat Au, (d) NpAu1 and (f) NpAu_4_. Partial current density for HER as obtained from the ionic current at *m*/*z* 2 by using eqn (3) with 0.5 atm. CO_2_ in 0.1 M NaHCO_3_ plus 0.4 M NaClO_4_ containing electrolyte (red) and in 0.1 M NaHCO_3_ plus 2 M NaClO_4_ containing electrolyte (orange) on (c) Flat Au, (e) NpAu1 and (g) NpAu 4. Scan rate: 5 mV s^−1^ and flow rate: 300 μL min^−1^.

This is understandable because the thin porous channels of NpAu catalysts generate an additional resistance that scales with increasing pore length.^43,44^ This is because the distance that the ions have to travel, will increase with increasing pore length. Hence, in going from NpAu1 to NpAu_4_ as the length of the nanoporous channels increases, the ohmic drop losses will also increase. As shown in Fig. 4a, at the orifices of the porous sample an ohmic drop originates solely due to the electrolyte resistance (*R*_sol_) which can be mitigated by positive feedback compensation. However, the distributed capacitance (*C*) and the charge transfer resistance (*R*_ct_) that represent the electrocatalytic reaction at the interface are coupled to additional uncompensated resistance (*r*_pore_) along the length of the pores. This means that there is an additional barrier for the current to pass through the pores. Hence, with increasing distance from the orifice it becomes more difficult for the porous channels to participate in the faradaic reaction. Essentially, these cumulative uncompensated ohmic drop effects can result in an electrocatalytically inactive zone at the bottom of the porous channels which results in the decoupling of the ECSA and the specific activity at these catalysts. Hence, the observed trend for the partial specific CO_2_RR current density as a function of the catalyst roughness (shown in Fig. 2b) can be attributed to the fact that the increasing availability of active sites for CO_2_RR from Flat Au to NpAu_4_ catalysts, is countered by a corresponding increase in the ohmic drop effects with increasing pore length. As a result, while at relatively low catalyst roughness (NpAu1; Fig. 2b) the increased availability of the active sites overshadows these ohmic drop effects, as the catalyst roughness (pore length) increases further and ohmic drop effects dominate the overall activity for CO_2_RR at NpAu catalysts.

In order to validate the presence of the ohmic drop effects at NpAu catalysts, we performed additional CO_2_RR measurements (shown in Fig. 4) by increasing the supporting electrolyte concentration in the system (at a fixed 0.1 M bicarbonate concentration). Since increasing supporting electrolyte concentration will increase the conductivity of the electrolyte, it should result in a proportional decrease in the ohmic drop (*r*_pore_) along the porous channels. Hence, we would expect the partial specific current density for CO_2_RR to increase with increasing ionic strength of the electrolyte at NpAu catalysts.

The results in Fig. 4 confirm this expectation: on all the NpAu catalysts (Fig. 4d and f) the partial specific current density for CO_2_RR increases with increasing concentration of the supporting electrolyte, while on flat Au catalysts, the partial current density for CO_2_RR slightly decreases with the increasing ionic strength (Fig. 4b). This confirms that ohmic drop effects along the porous channels play a significant role in determining the obtained specific current densities for CO_2_RR on NpAu catalysts. Notably, the presence of these ohmic drop effects on NpAu catalysts also implies even though the total amount of CO_2_ inside the pores increases with increasing pore size, this CO_2_ does not necessarily react everywhere within the pore. Because it takes longer for CO_2_ to diffuse out of a more porous electrode, this “unreacted” CO_2_ can lead to an enhanced current for CO_2_RR under non-steady-state conditions, until the pores are depleted of CO_2_ (as is the case in the results shown in Fig. 3b). Hence, it should be kept in mind that this local CO_2_ reservoir can affect the observed CO_2_RR activities on NpAu catalysts, however it is a non-steady-state effect.

Moreover, we note that unlike CO_2_RR, the HER activity increases with increasing concentration of the supporting electrolyte (Fig. 4c, e and f) regardless of the catalyst surface. This agrees with our previous studies on flat polycrystalline Au electrodes where we have shown that HER shows a positive reaction order for the cation concentration in the electrolyte. In that work we ascribed this to the changes in the double layer composition with changing cation concentration in the electrolyte.^33,41^ Moreover, in agreement with the current study, we also observed near-zero reaction order in cation concentration for CO_2_RR at flat polycrystalline Au electrodes, which we ascribed to the enhancement of HER at the expense of CO_2_RR with the increasing cation concentration in the electrolyte.^33^

**Fig. 5:**
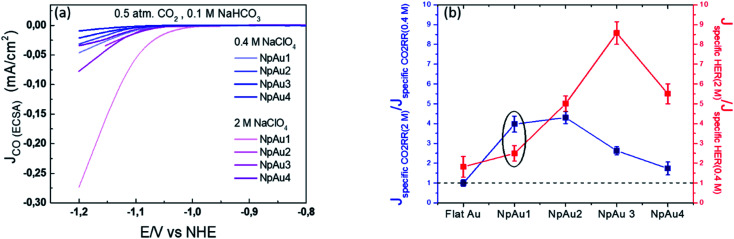
(a) Comparison of the partial specific current density for CO formation on different NpAu catalysts (NpAu_1_ to NpAu_4_) as obtained from the ionic current at *m*/*z* 28 by using eqn (3) with 0.5 atm. CO_2_ and 0.1 M NaHCO_3_ in 0.4 M NaClO4 (blue) and 2 M NaClO_4_ (pink) containing electrolyte at a scan rate of 5 mV s^−1^ and a flow rate of 300 μL min^−1^. (b) Ratio of the specific current density in 2 M NaClO_4_ containing electrolyte and 0.4 M NaClO_4_ containing electrolyte at a fixed NaHCO_3_ (0.1 M) concentration towards CO_2_RR (blue) and HER (red) at −1.2 V *vs.* NHE on different Au catalysts. Here a ratio greater than 1 indicates a higher specific current density in 2 M NaClO_4_ containing electrolyte compared to 0.4 M NaClO_4_ containing electrolyte.

**Fig. 6:**
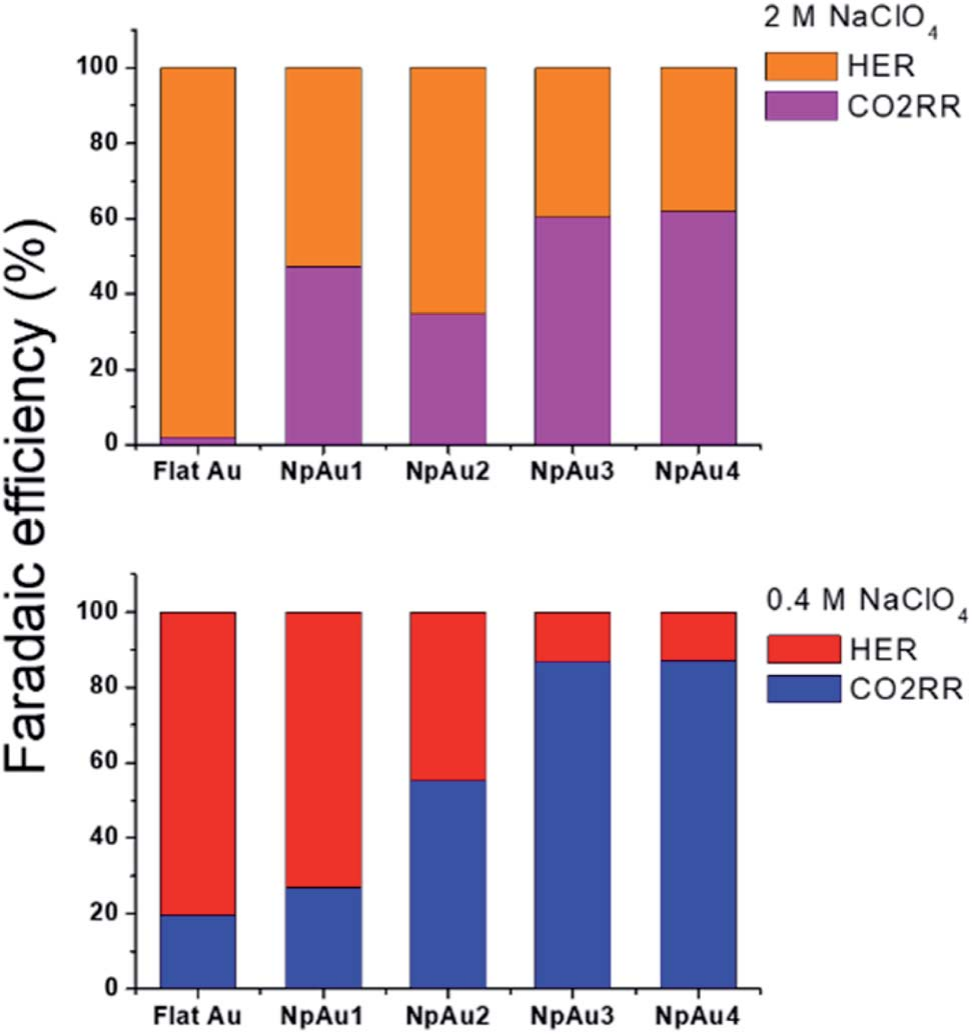
Comparison of the faradaic efficiency for CO_2_RR and HER in 2 M NaClO_4_ containing electrolyte (top panel) and 0.4 M NaClO_4_ containing electrolyte (bottom panel) at a fixed NaHCO_3_ concentration (0.1 M) at −1 V *vs.* NHE in 0.5 atm. CO_2_.

Additionally, in Fig. 5a we plot the partial specific current density for CO_2_RR on the different Au catalysts in 0.4 M and 2 M NaClO_4_ containing electrolyte and we see that while in the case of NpAu1, the increasing ionic strength of the electrolyte is able to compensate for the ohmic drop effects completely, for the other NpAu catalysts, only a slight improvement is observed. This shows that by increasing the total ionic strength of the electrolyte from 0.6 M to 2.1 M we are still not able to completely compensate for the ohmic drop effects, especially in catalysts with higher roughness factors. Nevertheless, increasing the ionic strength of the electrolyte does improve the obtained specific current density for CO_2_RR on all the NpAu catalysts. Importantly, there is a trade-off here between the increase in the CO_2_RR activity (specific current density) and CO_2_RR selectivity (faradaic efficiency) with the increasing ionic strength of the electrolyte. As shown in Fig. 5b, the enhancement in the HER activity with increasing ionic strength overshadows the enhancement in the CO_2_RR activity on all the catalysts except NpAu1. This is also reflected in the faradaic selectivity for CO_2_RR, as shown in Fig. 6, where we see that except NpAu1, in all the other NpAu catalysts, the faradaic selectivity towards CO_2_RR decreases with increasing ionic strength of the electrolyte. Hence, these results show that we can partially compensate for the ohmic drop effects occurring inside nanoporous catalysts by increasing the supporting electrolyte concentration which enables higher CO_2_RR activity. However, depending on the roughness/porosity of the catalysts, the concomitant enhancement of the competing HER reaction with increasing cation concentration in the electrolyte can overshadow the enhancement in the CO_2_RR activity, which may therefore result in a lower faradaic selectivity for CO_2_RR.

## 4. Conclusions

We studied the role of pore parameters (diameter and pore length) in tuning CO_2_RR on nanoporous Au (NpAu) catalysts by using the online DEMS technique. We find a higher CO_2_RR faradaic selectivity is achieved with increasingly narrower and longer pores (increasing catalyst roughness), and in the most extreme case (NpAu_4_) we achieve near 100% efficiency even in an under-saturated CO_2_ solution. This enhancement is attributed to two factors, the enhancement of the CO_2_RR currents due to the presence of more active sites with increasing catalyst roughness and the suppression of competing HER reaction due to the increasing local pH at the catalyst surface. Interestingly, the partial specific current density for CO_2_RR shows a more complicated relationship with catalyst roughness than the partial geometric current density, as it does not scale with increasing catalyst roughness. We show that this is due to the presence of additional ohmic drop across the length of the thin porous channels which plays an important role in determining the specific activity of nanoporous catalysts.

These results have important implications as they show that with increasing catalyst porosity, there is a trade-off between the number of active sites and the ohmic drop effects at the catalyst surface. These two effects counter-act each other for tuning the CO_2_RR activity. This is relevant as previous studies on nanoporous catalysts have not considered these offsets and some of the discrepancies in the current literature on the role of pore parameters in tuning CO_2_RR activity can be explained by the presence of these counter-acting effects.

In light of these results it will also be interesting to experimentally probe the local pH gradients at the surface of nanoporous catalysts as these results imply that the increase in the local alkalinity due to the generation of OH^−^ ions *via* CO_2_RR and HER, will diminish along the length of the pores due to the increasing ohmic drop effects. Hence, exclusion of these effects can lead to erroneous estimation/modelling of local pH gradients at the surface of nanoporous catalysts. However, the local measurement of pH is technically difficult as it is very sensitive to the pH probe used.^45,46^

In conclusion, this work provides important new insights into the intricate role of local pH gradients and intrinsic geometric effects in tuning the activity/selectivity of CO_2_RR at the surface of nonporous Au catalysts and we believe that these insights will be instrumental in the rational optimization of CO_2_RR on practical electrode geometries.

## Data availability

The datasets supporting this article have been uploaded as part of the ESI.†

## Author contributions

The manuscript was written through contributions of all authors. All authors have given approval to the final version of the manuscript.

## Conflicts of interest

There are no conflicts to declare.

## Supplementary Material

SC-013-D1SC05743J-s001
