# Supplementary material for: Effect of pore diameter and length on electrochemical CO2 reduction reaction at nanoporous gold catalysts
Source: Chem Sci. 2022 Feb 22;13(11):3288–98. doi: 10.1039/d1sc05743j (PMC8926346; doi:10.1039/d1sc05743j)
Supplement: SC-013-D1SC05743J-s001 [file SC-013-D1SC05743J-s001.pdf]

Electronic Supplementary Material (ESI) for Chemical Science.
This journal is © The Royal Society of Chemistry 2022

Supporting Information to

Effect of pore diameter and length on electrochemical CO_2 reduction reaction at nanoporous gold catalysts

Akansha Goyal[a], Christoph J. Bondue[a,b], Matthias Graf[a] and Marc T.M. Koper[a*]

[a]Leiden Institute of Chemistry, Leiden University, PO Box 9502, 2300 RA Leiden, The Netherlands

[b]Faculty of Chemistry and Biochemistry, Ruhr University Bochum, Bochum D-44780, Germany

* Corresponding author: m.koper@chem.leidenuniv.nl

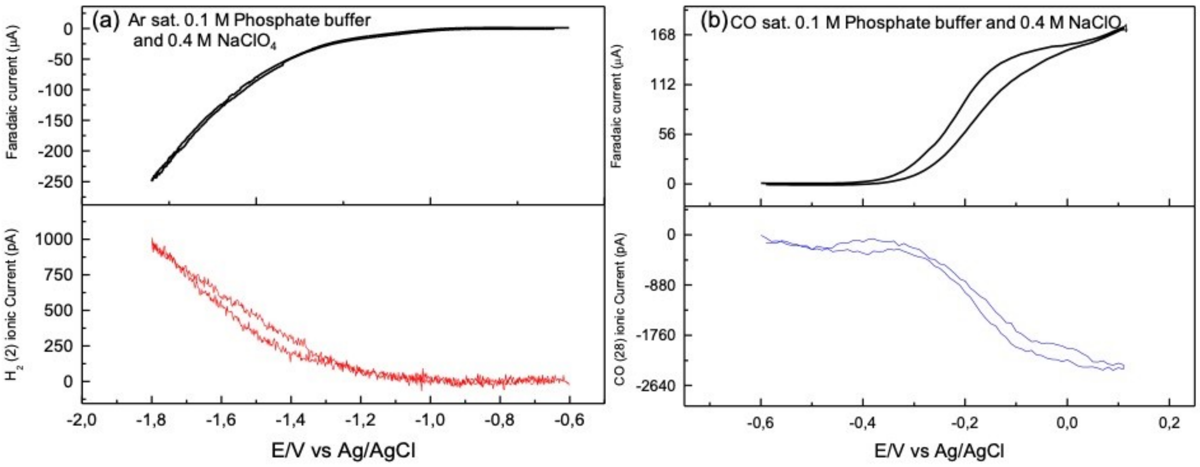

Figure S1 DEMS calibration experiment for (a) H_2 and (b) CO, performed in 0.1 phosphate buffer and 0.4 M $NaClO_4$ at 5 mVs^{-1} and a flow rate of 300 $\mu Lmin^{-1}$ in Ar sat. and CO sat. conditions respectively. For determining $K^{\circ}_{H_2}$ the Faradaic current for HER and the ionic current at m/z 2 as shown in S1a in the top panel and the bottom panel, respectively, were plugged into eqn. 3 (see Section 2.4 in the Main Manuscript) where z was taken to be 2. For determining K°_{CO} the Faradaic current for CO oxidation and the ionic current at m/z 28 as shown in S1b in the top panel and the bottom panel, respectively, were plugged into eqn. 3 (see Section 2.4 in the Main Manuscript) where z was taken to be 2.

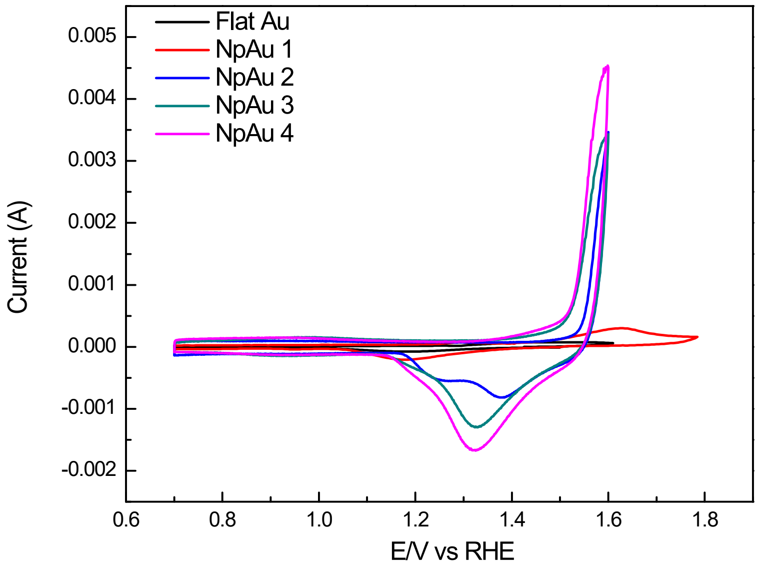

Figure S2 Characterization cyclic voltammograms for different Au catalysts in 0.1 M H_2SO_4 in Ar sat. environment at 50 mVs^{-1} where the charge of the Au_xO_y reduction peak was used to calculate the electrochemically active surface area (ECSA) of the different samples.

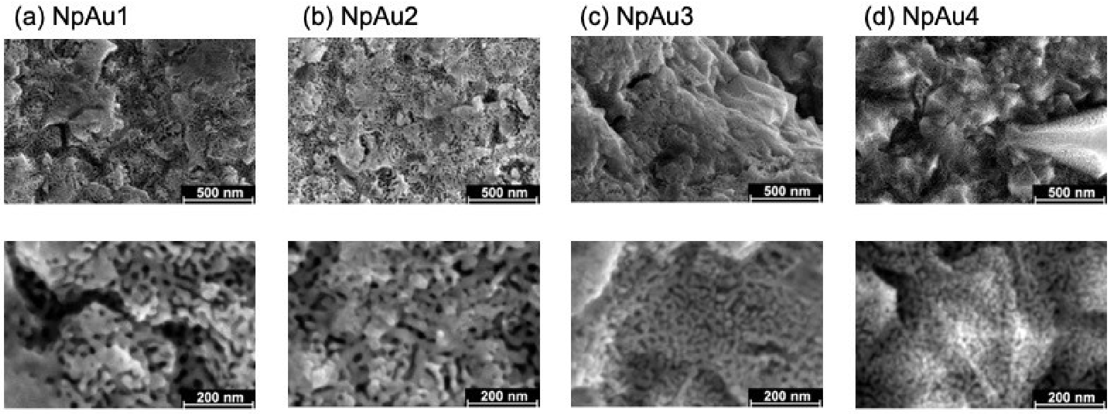

X_{Ag} (atomic %)	7.68±1.11	8.03±1.21	7.31±1.25	6.82±1.09

Figure S3 SEM characterization of different nano-porous Au (NpAu) samples, left to right: (a) NpAu1, (b) NpAu2, (c) NpAu3 and (d) NpAu4 where the upper row shows the low magnification SEM images and the lower row shows the high magnification SEM images for the different samples. At the bottom, the residual Ag content (X_{Ag}) as determined by the EDX measurements at low magnification is given for every NpAu sample along with the internal quantification error.

S1. Estimation of diffusion layer thickness, theoretical limiting current

To calculate the diffusion layer thickness, the ferricyanide-ferrocyanide redox couple was employed (shown in Fig. S4), where the diffusion limited current ($J_{limiting}$) obtained at -0.15 V (vs Ag/AgCl) was used to calculate the diffusion layer thickness by using Fick's first law of diffusion:

$$\delta = \frac{nFD_{Fe(CN_6)^{3-}} C^{o}_{K_3Fe(CN)_6}}{J_{limiting}} \qquad (S1)$$

Where n is no. of electrons involved in the reaction (i.e. n =1) F is Faraday's constant (96485 Cmol^{-1}), $D^{Fe(CN_6)^{3-}}$ is the diffusion coefficient of ferricyanide (7×10^{-6} cm^{2}s^{-1}), $C^{o}_{K_3Fe(CN)_6}$ is the bulk concentration of potassium ferricyanide (10 mM). The values obtained for the diffusion layer thickness in the dual layer thin cell geometry at different electrolyte flow rates are given in Table S1. Moreover, based on the diffusion layer thickness values, the mass transported limited current densities (calc. by using eqn. 1), both for CO2RR and bicarbonate-mediated HER are also calculated in Table S1.

We note that the partial currents due CO2RR and HER remain far below the theoretically calculated mass transport limiting currents calculated by using bulk concentrations of the reacting species (see column 4 of Table S1) under the experimental conditions of our measurements (electrolyte flowrate of 300 µL min^{-1}; experimental data shown in Fig. 1 of the main manuscript).

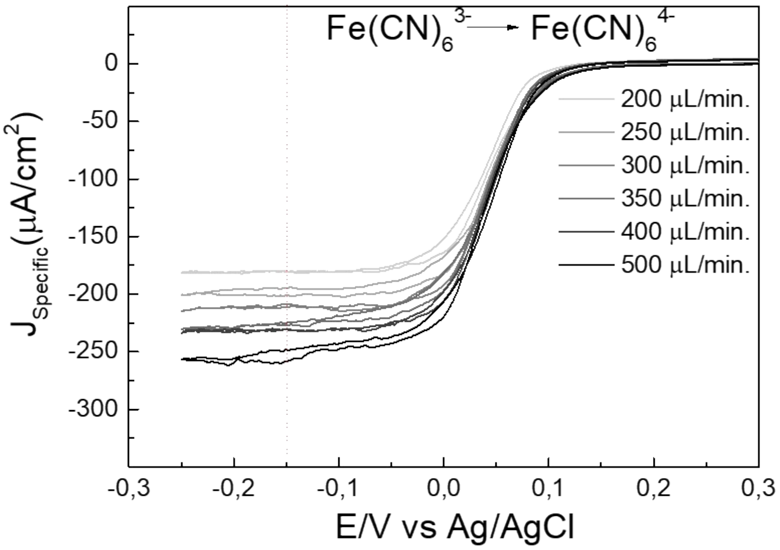

Figure S4 Determination of the diffusion layer thickness in the dual thin layer cell: cyclic voltammograms obtained in 0.1 M $NaHCO_3$ and 0.4 M $NaClO_4$ with 10 mM $K_3Fe(CN)_6$ at a scan rate of 5 mVs^{-1}, at different flow rates, where the diffusion limited currents for ferricyanide reduction at -0.15 V vs Ag/AgCl (indicated by the dotted line) were used to calculate the diffusion layer thickness by using Fick's law.

Flow rate (μL/min)	Diffusion layer thickness	$J_{CO2RR\ Limiting(theo.)}$ (mA/cm^2) at	$J_{HER\ Limiting(theo.)}$ (mA/cm^2) in 0.1 M $NaHCO_3$

	(μm)	0.5 atm. CO_2	
200	374.8	-1.40	-6.17
250	336.1	-1.56	-6.89
300	323.7	-1.62	-7.15
350	297.2	-1.76	-7.79
400	293.2	-1.79	-7.89
500	261.4	-2.01	-8.85

Table S1 Diffusion layer thickness (middle column) at different flow rates as calculated from the data shown in Figure S4 by using Fick's first law of diffusion, where no. of electrons involved were taken to be 1, concentration of $K_3Fe(CN)_6$ was taken to be 10 mM, diffusion coefficient of $Fe(CN)_6^{3-}$ was taken to be 7 $\times$ 10^{-6} cm^2s^{-1} and Faraday's constant was taken to be 96485 $Cmol^{-1}$. Thereafter, the theoretical diffusion limited current for CO2RR and bicarbonate mediated HER were calculated under the condition of our studies by again using Fick's law where the no. of electrons involved in the reaction was taken to be 2 and the concentration of CO_2 (aq.) was taken to be 16.5 mM (0.5 atm. CO_2), the concentration of HCO_3^- was taken to be 0.1 M, the diffusion coefficient of CO_2 was taken to be 1.6 $\times$ 10^{-5} cm^2/s, the diffusion coefficient of HCO_3^- was taken to be 1.2 $\times$ 10^{-5} cm^2/s and the values for the diffusion layer thickness were taken from the middle column of the table.

S2. Estimation of the local pH and local bicarbonate ion concentration

Both CO2RR as well as bicarbonate mediated HER lead to an increase in the local pH at the electrode surface, as the former leads to the generation of hydroxyl ions, while the latter leads to the consumption of the proton donor species i.e. bicarbonate ions. Then under the steady-state conditions, we can consider that the amount of hydroxyl ions generated and the amount of protons (i.e. bicarbonate ions) consumed will correspond to their mass transport flux to the surface. Hence, we obtain the following eqn. for j_{tot}:

$$j_{tot} = j_{HER} + j_{CO2RR}$$

$$= k_{eff}^{HCO_3^-} F\left(\left[HCO_3^-\right]_{bulk} - \left[HCO_3^-\right]_{surf}\right) + k_{eff}^{OH^-} F\left(\left[OH^-\right]_{Surf} - \left[OH^-\right]_{bulk}\right) \quad \text{(S2)}$$

Where k_{eff} is the effective mass transfer coefficient of HCO_3^- ions and OH^- ions such that $k_{eff} = \frac{D_{eff}}{\delta_{eff}}$ where D_{eff} is the effective diffusion coefficient which depends on the porosity of the catalyst i.e. $D_{eff} = P'D$. Here, P' is porosity of the catalyst corrected by its tortuosity (τ) i.e. $P' = \frac{P}{\tau}$ where $\tau = P^{-1/2}$ as given by Bruggeman correlation and *D* is the diffusion coefficient of the species in the solution phase.[1] Hence, with increasing coarsening, the effective diffusion coefficient increases, due to the increasing porosity of the catalyst.[1] And changes in the catalyst porosity lead to corresponding changes in the concentration gradients of these species at the electrode surface. Based on previous studies,[1] we will assume here that P' (corrected porosity) of our catalysts varies between 0.2 to 0.8 in going from the least coarsened sample to the most coarsened sample i.e. with decreasing surface roughness. We

note that these are two extreme values and, in the samples used in our studies, we may have different porosities than these values. However, we will take these two extremes as representative of the least coarsened sample (NpAu4) and the most coarsened sample (NpAu1) in our study, respectively. Moreover, we assume that the effective diffusion layer thickness (δ_{eff}) is essentially the length of the NpAu sample corrected by its tortuosity (δ_{eff} = $L\,\tau$; where L is the length of the porous channel i.e. thickness of the catalyst layer).[2] Since we do not have the precise value of pore length of our catalysts, here we will assume that it varies between 250 μm to 200 μm in going from the least coarsened sample (P' = 0.2) to the most coarsened sample (P' = 0.8). We note that based on previous studies, we would expect our pore length to be a bit shorter than 200 μm.[3] Here we use slightly higher values of pore length to see if we will reach mass transport limitations on nanoporous catalysts under more extreme conditions.

For the solution phase reactions, we will only consider the homogeneous equilibria between bicarbonate, hydroxyl and carbonate ions i.e. $HCO_3^- + OH^- \leftrightarrow CO_3^{2-} + H_2O$. This is a reasonable assumption because the rate of homogeneous HCO_3^- consumption is much faster than the rate of homogenous CO_2 consumption.

Then by considering that the homogeneous reaction between one ion of HCO_3^- and OH^- yields one ion of CO_3^{2-} and given that bicarbonate and carbonate have nearly identical diffusion coefficients ($D_{CO_3^{2-}}$ = 1.6 × 10^{-5} cm^2/s; $D_{HCO_3^-}$ = 1.2 × 10^{-5} cm^2/s), we can assume the concentration gradient between surfaces and bulk electrolyte is nearly identical for bicarbonate and carbonate, respectively. This translates into eqn. S3, which states that the surface concentration of carbonate $[CO_3^{2-}]_{surf}$ equals the bulk concentration of bicarbonate $[HCO_3^-]_{bulk}$ minus the surface concentration of bicarbonate $[HCO_3^-]_{surf}$ (we consider that the bulk concentration of carbonate is negligible):

$$[CO_3^{2-}]_{surf} = [HCO_3^-]_{bulk} - [HCO_3^-]_{surf} \qquad \text{(S3)}$$

Rearrangement of eqn. S3 and condsidering the homogeneous equilibria of HCO_3^- ($HCO_3^- + OH^- \leftrightarrow CO_3^{2-} + H_2O$; K_{eq}= 4.8 × 10^3 L mol^{-1}S^{-1}) yields eqn. S4, which gives the following expression for the surface concentration of bicarbonate ions:

$$[HCO_3^-]_{surf} = \frac{[HCO_3^-]_{bulk}}{1 + K_{eq}[OH^-]_{surf}} \qquad \text{(S4)}$$

By substituting the expression for $[HCO_3^-]_{surf}$ from eqn. S4 into eqn. S2 we get:

$$j_{tot} = k_{eff}^{HCO_3^-} F\left([HCO_3^-]_{bulk} - \frac{[HCO_3^-]_{bulk}}{1 + K_{eq}[OH^-]_{surf}}\right) + k_{eff}^{OH^-} F\left([OH^-]_{Surf} - [OH^-]_{bulk}\right) \qquad \text{(S5)}$$

The above eqn. can be rearranged to get a quadratic eqn. for $[OH^-]_{Surf}$which can be solved as follows:

$$[OH^-]_{Surf} = \frac{-b \pm \sqrt{b^2 - 4ac}}{2a} \qquad \text{(S6)}$$

where

$a = K_{eq} k_{eff}^{OH^-}$

$$b = K_{eq}k_{eff}^{HCO_3^-}[HCO_3^-]_{bulk} + k_{eff}^{OH^-} - K_{eq}k_{eff}^{OH^-}[OH^-]_{bulk} - \frac{j_{tot}K_{eq}}{F}$$

$$c = k_{eff}^{OH^-}[OH^-]_{bulk} - \frac{j_{tot}}{F}$$

In Fig. S5 we have calculated the surface pH (eqn. S6) as well as the value of $[HCO_3^-]_{surf}$ (eqn. S4) at 3 different catalyst porosities ($P' = 0.8, 0.5, 0.2$), where the catalyst roughness increases with decreasing porosity. Moreover, we use the j_{tot} (total geometric current density) value obtained in Fig. 1d (main manuscript) on flat Au catalysts for all the calculations. We note that our experimental measurements have shown that the total geometric current density remains unperturbed with changing catalyst roughness, it's only the ratio of J_{HER} and J_{CO2RR} that changes. Hence, in our calculations, the changes in the local concentration gradients with changing catalyst roughness are essentially accounted for by the changing effective mass transfer coefficient of bicarbonate and OH^- with changing catalyst porosity.

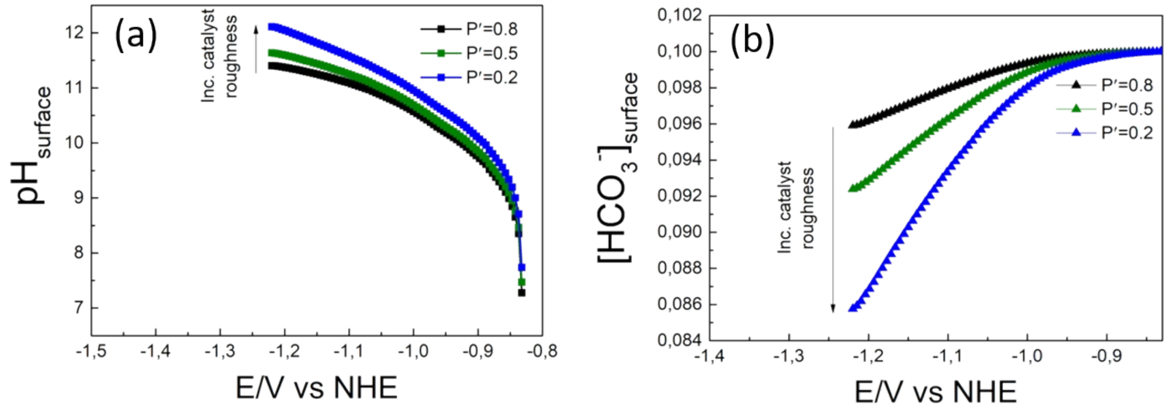

Figure S5 Calculated (a)Surface pH (b) $[HCO_3^-]_{surf}$ as a function of applied potential (vs RHE) as obtained from eqn. S6 and S4 respectively. Here j_{tot} was taken from the experimentally obtained current densities shown in Fig. 1 (main manuscript) and δ was taken to be 323.7 μm (corresponds to the electrolyte flow rate of 300 μL/min which is used in our experiments). The changing porosity of the catalyst surface was used to calculate the effective diffusion coefficients of bicarbonate and OH^- such that $D_{eff} = P'D$ where $D_{HCO_3^-}$ was taken to be 1.2 × 10^{-5} cm^2/s and D_{OH^-} was taken to be 5.27 × 10^{-5} cm^2/s. A decreasing corrected porosity P' represents an increasing catalyst roughness.

In Fig. S5 we see that the surface pH is more alkaline than the bulk pH (bulk pH = 7), irrespective of the catalyst porosity/roughness. Moreover, as expected, as the catalyst roughness increases (decreasing porosity P') the surface pH shows a corresponding increase. At the most negative applied overpotential (-1.25 V vs RHE) the surface pH increases from 11.2 (for P'=0.8) to 12 (for P'=0.2). Hence the surface concentration of hydroxyl ions increases from 1.9 × 10^{-3} M to 1 × 10^{-2} M in going from the least rough catalyst to the roughest catalyst. Moreover, this increase in the surface concentration of hydroxyl ions leads to a corresponding decrease in the surface concentration of bicarbonate ions such that it decreases from 0.095 M for P'=0.8 to 0.085 M for P'=0.2. And even though the surface concentration of bicarbonate ions is decreasing with increasing catalyst roughness, they are not yet entirely depleted at the surface. Moreover, in Fig. S6 we show the calculated current for HER with different catalyst porosities, and in agreement with the experimental results shown in Fig. 1e of the main manuscript, we see a suppression in the HER current with increasing catalyst roughness. Hence, there is a qualitative agreement between our experimental results and the calculated current densities for HER.

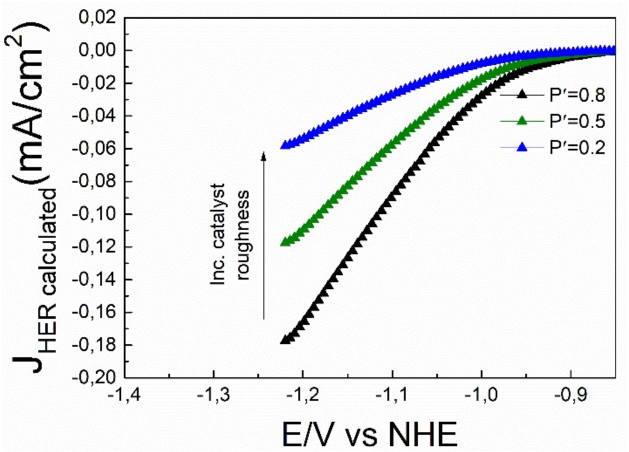

Figure S6 Calculated j_{HER} as obtained by eqn. S2, where the surface concentration of bicarbonate ions was taken from Fig. S5b and the bulk concentration of bicarbonate ions was taken to be 0.1 M. The diffusion layer thickness was δ was taken to be 323.7 μm (corresponds to the electrolyte flow rate of 300 μL/min which is used in our experiments). The changing porosity of the catalyst surface was used to calculate the effective diffusion coefficients of bicarbonate such that $D_{eff} = P^{'}D$ where $D_{HCO_3^-}$ was taken to be 1.2 × 10^{-5} cm^2/s and D_{OH^-} was taken to be 5.27 × 10^{-5} cm^2/s. A decreasing corrected porosity $P^{'}$ represents an increasing catalyst roughness.

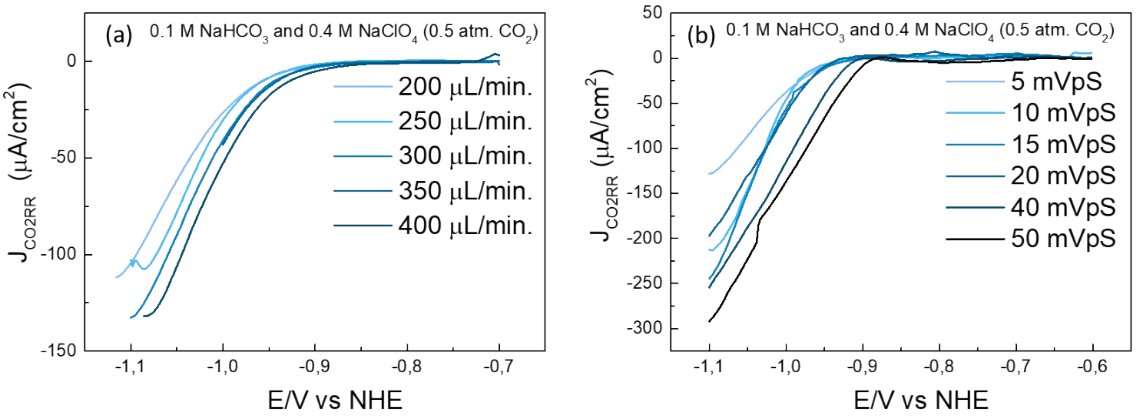

Figure S7 Partial current density for CO formation on Flat Au as obtained from the ionic current at m/z 28 by using eqn. 3 (in the main manuscript) with 0.5 atm. of CO_2 in 0.1 M $NaHCO_3$ and 0.4 M $NaClO_4$ containing electrolyte at (a) different flow rates and (b) different scan rates.

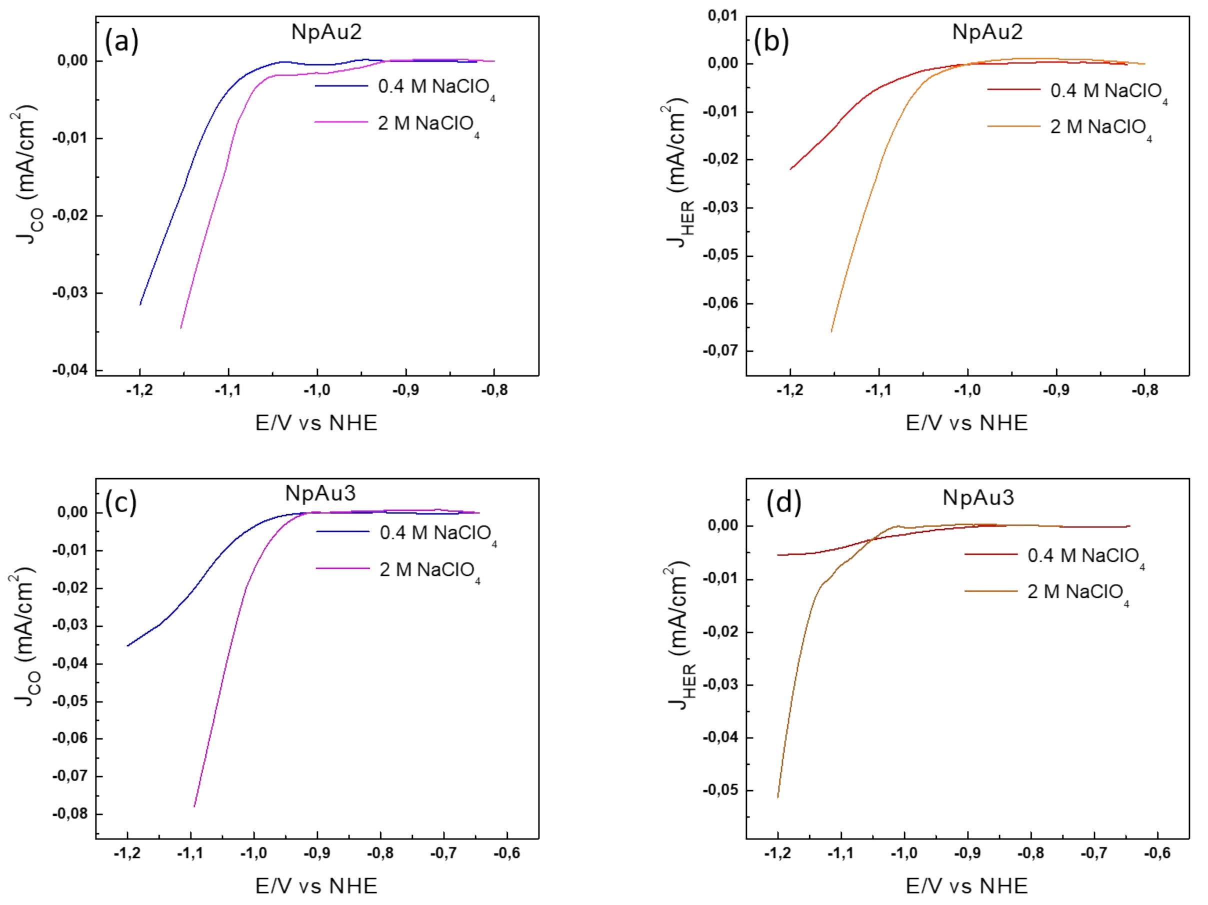

Figure S8 Partial specific current density for CO formation as obtained from the ionic current at m/z 28 by using eqn. 3 (Main Manuscript) with 0.5 atm. CO_2 in 0.1 M $NaHCO_3$ and 0.4 M $NaClO_4$ containing electrolyte (blue) and in 0.1 M $NaHCO_3$ and 2 M $NaClO_4$ containing electrolyte (purple) at a scan rate of 5 mVs^{-1} and a flow rate of 300 $\mu Lmin^{-1}$ on (a) NpAu2 and (c) NpAu3 and Partial current density for HER as obtained from the ionic current at m/z 2 by using eqn. 3 (Main Manuscript) with 0.5 atm. CO_2 in 0.1 M $NaHCO_3$ and 0.4 M $NaClO_4$ containing electrolyte (red) and in 0.1 M $NaHCO_3$ and 2 M $NaClO_4$ containing electrolyte (orange) at a scan rate of 5 mVs^{-1} and a flow rate of 300 $\mu Lmin^{-1}$ on (b) NpAu2 and (d) NpAu 3.

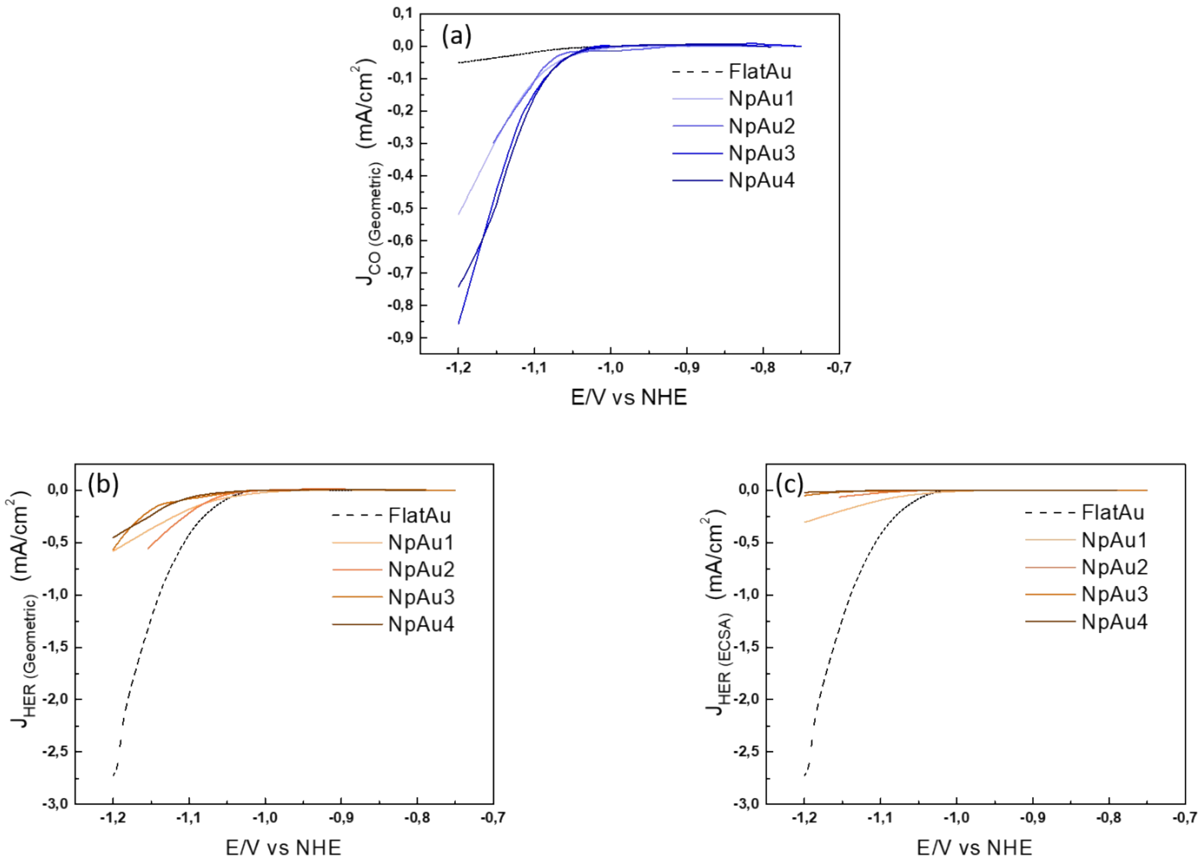

Figure S9 (a) Partial geometric current density for CO formation on different Au catalysts as obtained from the ionic current at m/z 28 by using eqn. 3 (Main Manuscript) with 0.5 atm. CO_2 in 0.1 M $NaHCO_3$ and 2 M $NaClO_4$ containing electrolyte at a scan rate of 5 mVs^{-1} and a flow rate of 300 $\mu Lmin^{-1}$. (b) Partial geometric current density for HER and (c) Partial specific current density for HER, as obtained from the ionic current at m/z 2 by using eqn. 3 (Main Manuscript) with 0.5 atm. CO_2 in 0.1 M $NaHCO_3$ and 2 M $NaClO_4$ containing electrolyte at a scan rate of 5 mVs^{-1} and a flow rate of 300 $\mu Lmin^{-1}$.

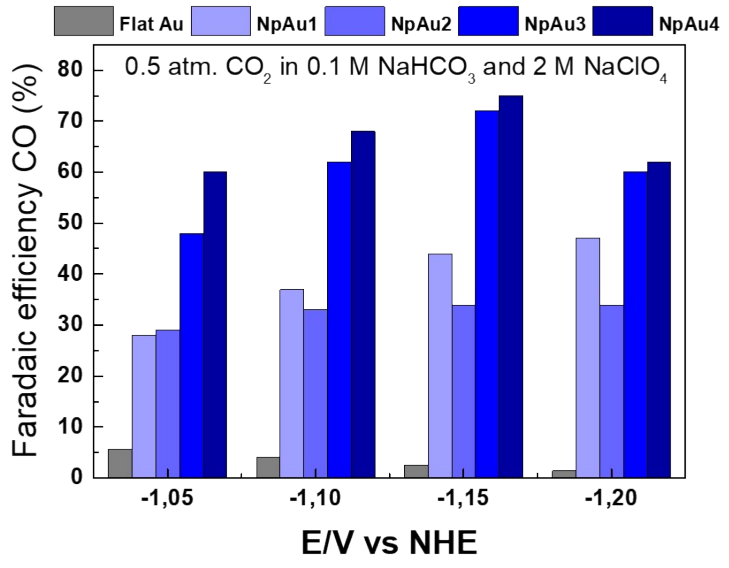

Figure S10 Faradaic efficiency for CO formation of the different Au catalysts in 0.5 atm. CO_2 and 0.1 M $NaHCO_3$, 2 M $NaClO_4$ containing electrolyte at different potentials (vs. NHE) as obtained from the DEMS measurements by using eqn. 4 (Main Manuscript).

References

1. Haensch, M.; Balboa, L.; Graf, M.; Silva Olaya, A. R.; Weissmüller, J.; Wittstock, G., Mass Transport in Porous Electrodes Studied by Scanning Electrochemical Microscopy: Example of Nanoporous Gold. *ChemElectroChem* **2019,** *6* (12), 3160-3166.
2. Johansson, M. V.; Testa, F.; Perrier, P.; Vicente, J.; Bonnet, J. P.; Moulin, P.; Graur, I., Determination of an effective pore dimension for microporous media. *International Journal of Heat and Mass Transfer* **2019,** *142*, 118412.
3. Graf, M.; Jalas, D.; Weissmüller, J.; Petrov, A. Y.; Eich, M., Surface-to-Volume Ratio Drives Photoelelectron Injection from Nanoscale Gold into Electrolyte. *ACS Catalysis* **2019,** *9* (4), 3366-3374.
